# Supplementary material for: Getting More Out of Biomedical Documents with GATE's Full Lifecycle Open Source Text Analytics
Source: PLoS Comput Biol. 2013 Feb 7;9(2):e1002854. doi: 10.1371/journal.pcbi.1002854 (PMC3567135; doi:10.1371/journal.pcbi.1002854)
Supplement: Dataset S1 — GATE software. Dataset S1 bundled with this paper contains a distribution of GATE (or see http://gate.ac.uk/download/). (TGZ) [file pcbi.1002854.s001.tgz › plos-gate/doc/announce-3-1.html]

GATE Version 3.1 release (April 2006)


# GATE Version 3.1 release (April 2006)

# 1. Major new features

## 1.1. Support for UIMA

UIMA (http://www.research.ibm.com/UIMA/) is a language processing framework
developed by IBM. UIMA and GATE share some functionality but are
complementary in most respects. GATE now provides an interoperability layer
to allow UIMA applications to include GATE components in their processing
and vice-versa. For full information, see chapter 14 of the User Guide.

## 1.2. New Ontology API

The ontology layer has been rewritten in order to provide an abstraction
layer between the model representation and the tools used for input and
output of the various representation formats. An implementation that uses
Jena 2 (http://jena.sourceforge.net/ontology) for reading and writing OWL
and RDF(S) is provided.

## 1.3. Ontotext Japec Compiler

Japec is a compiler for JAPE grammars developed by Ontotext Lab. It has some
limitations compared to the standard JAPE transducer implementation, but can
run JAPE grammars up to five times as fast. By default, GATE still uses the
stable JAPE implementation, but if you want to experiment with Japec, see
section 9.27 of the User Guide.

# 2. Other new features and improvements

- Addition of a new JAPE matching style "all". This is similar to Brill, but
  once all rules from a given start point have matched, the matching will
  continue from the next offset to the current one, rather than from the
  position in the document where the longest match finishes. More details can
  be found in Section 7.2.
- Limited support for loading PDF and Microsoft Word document formats. Only
  the text is extracted from the documents, no formatting information is
  preserved.
- The Buchart parser has been deprecated and replaced by a new plugin called
  SUPPLE - the Sheffield University Prolog Parser for Language Engineering.
  Full details, including information on how to move your application from
  Buchart to SUPPLE, is in section 9.12.
- The Hepple POS Tagger is now open-source. The source code has been
  included in the GATE distribution, under src/hepple/postag. More
  information about the POS Tagger can be found in Section 8.4.
- Minipar is now supported on Windows. minipar-windows.exe, a modified
  version of pdemo.cpp is added under the gate/plugins/minipar directory to
  allow users to run Minipar on windows platform. While using Minipar on
  Windows, this binary should be provided as a value for miniparBinary
  parameter. For full information on Minipar in GATE, see section 9.10 of
  the User Guide.
- The XmlGateFormat writer(Save As Xml from GATE GUI, gate.Document.toXml()
  from GATE API) and reader have been modified to write and read GATE
  annotation IDs. For backward compatibility reasons the old reader has been
  kept. This change fixes a bug which manifested in the following situation:
  If a GATE document had annotations carrying features of which values were
  numbers representing other GATE annotation IDs, after a save and a reload
  of the document to and from XML, the former values of the features could
  have become invalid by pointing to other annotations. By saving and
  restoring the GATE annotation ID, the former consistency of the GATE
  document is maintained. For more information, see Section 6.5.2 of the
  User Guide.
- The NP chunker and chemistry tagger plugins have been updated. Mark
  Greenwood has relicenced them under the LGPL, so their source code has
  been moved into the GATE distribution. See sections 9.3 and 9.15 for
  details.
- The Tree Tagger wrapper has been updated with an option to be less strict
  when characters that cannot be represented in the tagger's encoding are
  encountered in the document. Details are in section 9.7.
- JAPE Transducers can be serialized into binary files. The option to load
  serialized version of JAPE Transducer (an init-time parameter
  binaryGrammarURL) is also implemented which can be used as an alternative
  to the parameter grammarURL. More information can be found in Section 7.7.
- On Mac OS, GATE now behaves more naturally. The application menu items
  and keyboard shortcuts for About and Preferences now do what you would
  expect, and exiting GATE with command-Q or the Quit menu item properly
  saves your options and current session.
- Updated versions of Weka (3.4.6) and Maxent (2.4.0).
- Optimisation in gate.creole.ml: the conversion of AnnotationSet into ML
  examples is now faster.
- It is now possible to create your own implementation of Annotation, and
  have GATE use this instead of the default implementation. See
  AnnotationFactory and AnnotationSetImpl in the gate.annotation package for
  details.

# 3. Bug fixes

- The Tree Tagger wrapper has been updated in order to run under Windows.
  See 9.7.
- The SUPPLE parser has been made more user-friendly. It now produces more
  helpful error messages if things go wrong. Note that you will need to
  update any saved applications that include SUPPLE to work with this
  version - see section 9.12 of the User Guide for details.
- Miscellaneous fixes in the Ontotext JapeC compiler.
- Optimization : the creation of a Document is much faster.
- Google plugin: The optional pagesToExclude parameter was causing a
  NullPointerException when left empty at run time. Full details about the
  plugin functionality can be found in section 9.20.
- Minipar, SUPPLE, TreeTagger: These plugins that call external processes
  have been fixed to cope better with path names that contain spaces. Note
  that some of the external tools themselves still have problems handling
  spaces in file names, but these are beyond our control to fix. If you want
  to use any of these plugins, be sure to read the documentation to see if
  they have any such restrictions.
- When using a non-default location for GATE configuration files, the
  configuration data is saved back to the correct location when GATE exits.
  Previously the default locations were always used.
- Jape Debugger: ConcurrentModificationException in JAPE debugger. The JAPE
  debugger was generating a ConcurrentModificationException during an attempt
  to run ANNIE. There is no exception when running without the debugger
  enabled. As result of fixing one unnesesary and incorrect callback to
  debugger was removed from SinglePhaseTransducer class.
- Plus many other small bugfixes...
